# Modeling the change in European and US COVID-19 death rates

**DOI:** 10.1371/journal.pone.0268332

**Published:** 2022-08-17

**Authors:** Zeina S. Khan, Frank Van Bussel, Fazle Hussain

**Affiliations:** Department of Mechanical Engineering, Texas Tech University, Lubbock, TX, United States of America; Chinese Academy of Medical Sciences and Peking Union Medical College, CHINA

## Abstract

Motivated by several possible differences in Covid-19 virus strains, age demographics, and face mask wearing between continents and countries, we focussed on changes in Covid death rates in 2020. We have extended our Covid-19 multicompartment model (Khan et al., 2020) to fit cumulative case and death data for 49 European countries and 52 US states and territories during the recent pandemic, and found that the case mortality rate had decreased by at least 80% in most of the US and at least 90% in most of Europe. We found that death rate decreases do not have strong correlations to other model parameters (such as contact rate) or other standard state/national metrics such as population density, GDP, and median age. Almost all the decreases occurred between mid-April and mid-June 2020, which corresponds to the time when many state and national lockdowns were relaxed resulting in surges of new cases. We examine here several plausible causes for this drop—improvements in treatment, face mask wearing, new virus strains, testing, potentially changing demographics of infected patients, and changes in data collection and reporting—but none of their effects are as significant as the death rate changes suggest. In conclusion, this work shows that a two death rate model is effective in quantifying the reported drop in death rates.

## Introduction

A novel strain of coronavirus, SARS-CoV-2, causing Covid-19 disease, was identified in December 2019 by Chinese Health authorities in the city of Wuhan (Hubei), China [1, 2]. This disease has spread worldwide and many governments instituted containment measures, including city and state lockdowns and prohibiting travel from affected areas. However, such restrictions are difficult to sustain in the long term, with millions of people being affected by poverty and unemployment [3]. As a result, many jurisdictions eased population restrictions as of May 2020 to lessen the economic impact of the disease [3–5]. The result in the US of states ending their lockdowns then was a surge of cases in the early summer of 2020 [6, 7]; this was followed in the fall of 2020 by an even more massive surge in both the US and Europe brought on by by the onset of cold weather. Towards the end of 2020 there arose contagious new strains of Covid-19 in the UK, California, South Africa, and Brazil; and starting in December of 2020 jurisdictions began the rollout of the Pfizer, Moderna, and other vaccines, which will hopefully bring the end of the pandemic in sight [8].

Surprisingly, despite the large increases in Covid-19 cases in the United States in the summer of 2020, the number of deaths due to this virus did not mirror the dramatically increased case counts. Though this trend was noted by political and health commentators [9–11], there were at the time few mentions of any change of death rates in the epidemiological and modeling literature; those that did were primarily studies tracking cases in specific hospital systems [12–15]. In November 2020 *Nature* published a news feature based on interviews with clinicians which affirmed that the case mortality rate of Covid-19 had in fact fallen [16]. The aim of this piece was not so much quantifying the phenomenon or determining the geographical extents as encouraging research into the cause, some of which we will look at in the Discussion and Conclusions section of this paper. The first published research paper to focus on measurements of the change of transmission and death rates used a piecewise linear fitting model to identify points in time where significant changes occurred [17]. This study, published in July 2020, found that death rates in France, Belgium, Germany, the Netherlands, Italy, Switzerland, and Great Britain decreased by approximately 97–99%, and death rates in the United States, Portugal, and Sweden decreased approximately 93–96% from early March to early May 2020. It is reasonable to expect significant differences in linear fit segments to case data and death curves, in comparison with our model fits that describe the disease very closely (see below) primarily due to inclusion of multiple compartments. A second published research paper to focus on measurements of the change in rate across multiple jurisdictions is [18], from March 2021, which uses standard statistical techniques applied to death and case counts for blocks of time lasting several months. They found that the median reduction in the case fatality rate was 38% for the 53 countries with the highest death tolls.

While one of the main applications of epidemiological models is making predictions, it is also common for models to be used to make measurements of past and current quantities of interest that can be derived from model parameters and solutions. For example, Gupta et al. [19] use a prediction model to compare the virulence of Covid-19 with several other diseases including MERS and Ebola, while Al Zobbi et al. [20] use a model of short term changes in $R_{0}$ to measure how well various jurisdictions implemented their lockdowns in April and May of 2020. Here we give measurements of the changes in the case mortality rate across entire jurisdictions for all US states plus Washington DC and Puerto Rico, and all European countries (except Russia) plus Turkey, made by fitting a compartmental ODE disease model to state and national case and death data. Our finding is that in most of these jurisdictions the death rate for diagnosed individuals decreased dramatically (≈ 80% in the US and 90% in Europe), and almost all jurisdictions had a decrease of at least 30%. These decreases happened largely in late April, May, and early June 2020 as many jurisdictions were easing lockdowns which resulted in surging cases before vaccination campaigns began; more recent refits have shown the lower mortality rates persisting into 2021. Having checked several quantitative regional factors that could influence these fatality rates, including basic age demographics, population density, geographical location, and certain economic indicators, we have not found strong correlations to the magnitude of the drop in death rate, or the initial or final death rates individually. Several plausible causes for this dramatic drop are examined in the Discussion, such as improvements in treatment, face mask wearing, testing, new virus strains, potentially changing demographics of infected patients, and revisions to data collection and reporting, but none alone convincingly explain the magnitude of change we have modeled given the currently available evidence.

## Materials and methods

### Model

**Fig 1:**
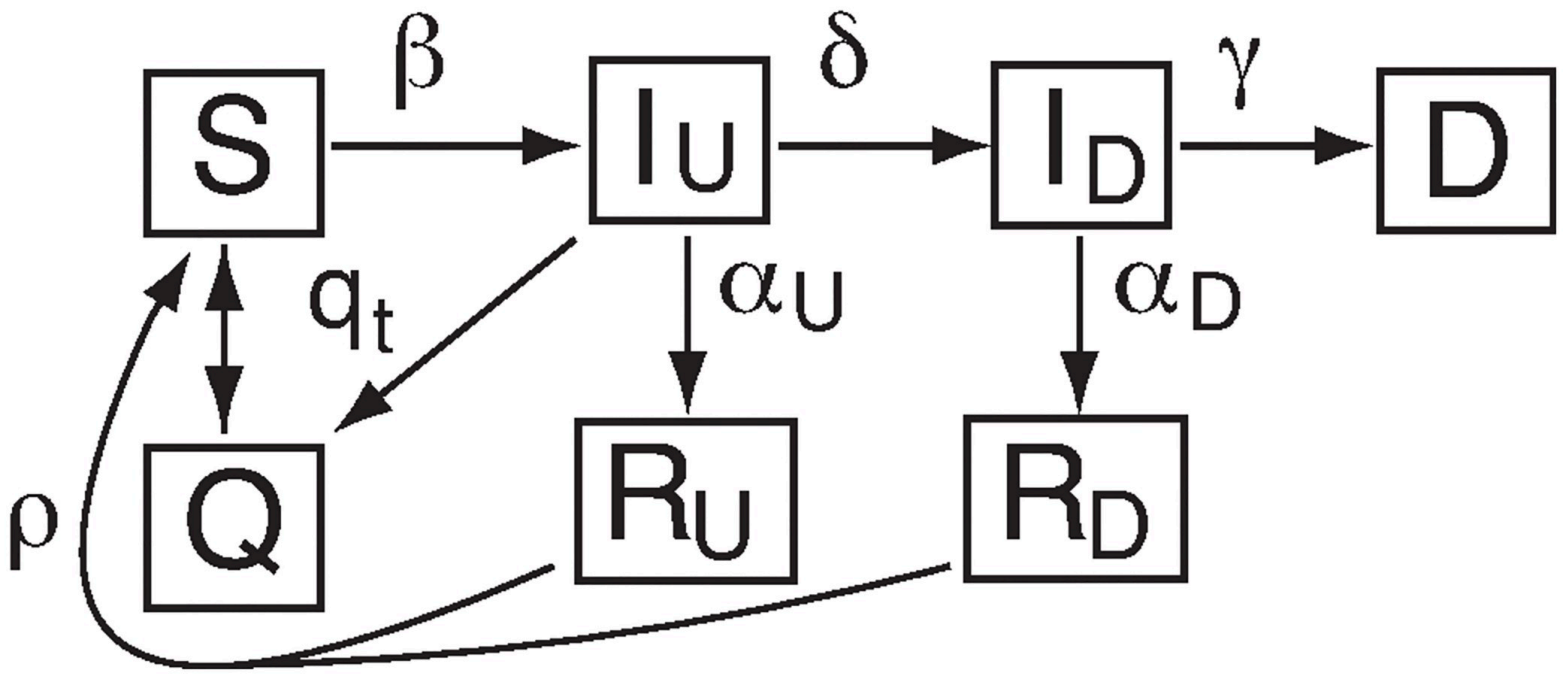
Schematic of the compartments. *S* is susceptible, *I*_*U*_ undetected infected, *I*_*D*_ detected infected, *D* detected deceased, *Q* sequestered, *R*_*U*_ undetected recovered, and *R*_*D*_ detected recovered. Transfer between compartments are indicated by arrows labelled with associated rate coefficients.

To calculate the change in death rate we used a slightly modified version of our compartment model, first presented in [21]. This is a SIR-based ODE model that includes extra compartments and transfer rates to deal with: detected versus undetected infecteds, isolation on diagnosis, effects of social distancing policies, and possible loss of immunity for recovered populations (both detected and undetected). The model thus requires splitting the infected and recovered compartments into detected and undetected components (respectively, *I*_*D*_ and *I*_*U*_, *R*_*D*_ and *R*_*U*_). As well, we include two new compartments: *Q* (pseudo-Quarantine, a bin that allows us to model the effect of social distancing policies, and *D*, for detected infecteds who die. This last compartment was not necessary for the model per se; deaths are not part of the basic SIR model, which is static, and we did not include a compartment for deaths of undetected infecteds either. However, deaths due to COVID-19 are a readily available statistic, and possibly more trustworthy than caseloads, so it was added for modeling/fitting purposes. Fig 1 shows the connections among different compartments in our model; the rate equations are as follows:

$$\begin{array}{c} \frac{dS}{dt}=-\beta S{I_{U}}^{a}-q_{t}S+\rho \left(R_{U}+R_{D}\right) \end{array}$$ (1)

$$\begin{array}{c} \frac{dI_{U}}{dt}=\beta S{I_{U}}^{a}-\left(q_{t}+\alpha_{U}+\delta \right)I_{U} \end{array}$$ (2)

$$\begin{array}{c} \frac{dI_{D}}{dt}=\delta I_{U}-\left(\gamma_{i}+\alpha_{D}\right)I_{D} \end{array}$$ (3)

$$\begin{array}{c} \frac{dR_{U}}{dt}=\alpha_{U}I_{U}-\rho R_{U} \end{array}$$ (4)

$$\begin{array}{c} \frac{dR_{D}}{dt}=\alpha_{D}I_{D}-\rho R_{D} \end{array}$$ (5)

$$\begin{array}{c} \frac{dD}{dt}=\gamma_{i}I_{D} \end{array}$$ (6)

$$\begin{array}{c} \frac{dQ}{dt}=q_{t}\left(S+I_{U}\right) \end{array}$$ (7)

If normalized quantities are used then the sum of all the compartments must always equal 1 (as in the SIR model). The coefficients *β* (transmissability), *δ* (detection rate), *α*_*U*_, *α*_*D*_ (recovery rates for undetected and detected infecteds respectively), *γ*_1_ and *γ*_2_ (death rates for detected infecteds), *ρ* (loss of immunity for recovered infecteds) and the exponent *a* are constants, which can be used as fit parameters. While this model could have many uses, our original purpose was to try to measure what proportion of infecteds were eventually being detected (our finding: about half in almost all jurisdictions).

In [21] we give a term-by-term explication of the equations; here we will restrict ourselves to mentioning two aspects of the model that are probably most unfamiliar to those who have encountered the SIR model and its variations before. First, the incidence rate $\beta S{I_{U}}^{a}$, the average normalized new infections in time, is nonlinear when the exponent *a* is close to 1. This is done to adjust for the effect of heterogeneous population densities. Second, *q*_*t*_ is the time dependent transfer rate from the *S* and *I*_*U*_ populations to the *Q* bin (and back, when negative). It should be kept in mind that the *Q* pseudo-quarantine does not imply that any group of undiagnosed people are physically sequestered anywhere; this is merely an attempt to represent the average reduction in daily contact people have during periods of lockdown and other social distancing efforts (medical quarantine will be dealt with in the next subsection). Alternatively, this might possibly be modeled by altering *β* or the power law dependency *a* in a time-dependent way; however, since *β* in particular represents the contagiousness of the pathogen as much as the sociability of the population, we thought it better to adjust potential contacts directly by adjusting populations (plus note, the three alternatives do not give mathematically equivalent results). In principle the population transfer could be done instantaneously, but for reasons of the implementation (to be discussed below) we use a rate that is 0 most of the time, increases rapidly around the specified time of transfer into *Q*, and decreases quickly again to 0 when the specified proportion of population has been transferred. Negative values of *q*_*t*_ specify transfer out of the *Q* bin back into the general population.

### Assumptions

To make the model tractable for implementation in an ODE solver several simplifying assumptions have been made. To start, in the model detected infecteds (*I*_*D*_) do not transmit the disease to the Susceptible (*S*) population. This not only reflects the very rigorous medical isolation protocols in place for hospitalized cases across the US, Europe, and the Pacific rim (and the hopeful but often unverified self-quarantine recommended for less-serious diagnosed cases), but also that reported numbers of transmissions from known cases seem to have been negligible compared to transmission from undiagnosed and asymptomatic infecteds (as well as things like faulty ventilation systems).

Further, even though we have incorporated detected deaths in the model (since, as stated above, this doubles the amount of input data that can be applied for fitting), we do not attempt to model undetected deaths, since there is no corresponding official data stream for this quantity originating with hospitals and coroners’ offices. While the results of excess death studies are scientifically interesting in their own right, they are too speculative and at too great a time lag to be a basis for the kind of fast-turnover modelling we are doing here. Similarly, we do not consider the effects of births, immigration, emigration, or deaths due to other diseases or trauma.

In the same spirit of keeping the model as simple as possible when data do not warrant additional elements, populations in the *R*_*U*_ and *R*_*D*_ compartments lose immunity at the same rate *ρ*. On the other hand, we have kept separate recovery rates for detected and undetected infecteds, since forcing a single rate would have the mathematical effect of creating a correlation between two distinct pathways in the model; while such a correlation may or may not exist, it is better to obtain it after the fact from the fit results by comparing the value of two different fit parameters.

**Fig 2:**
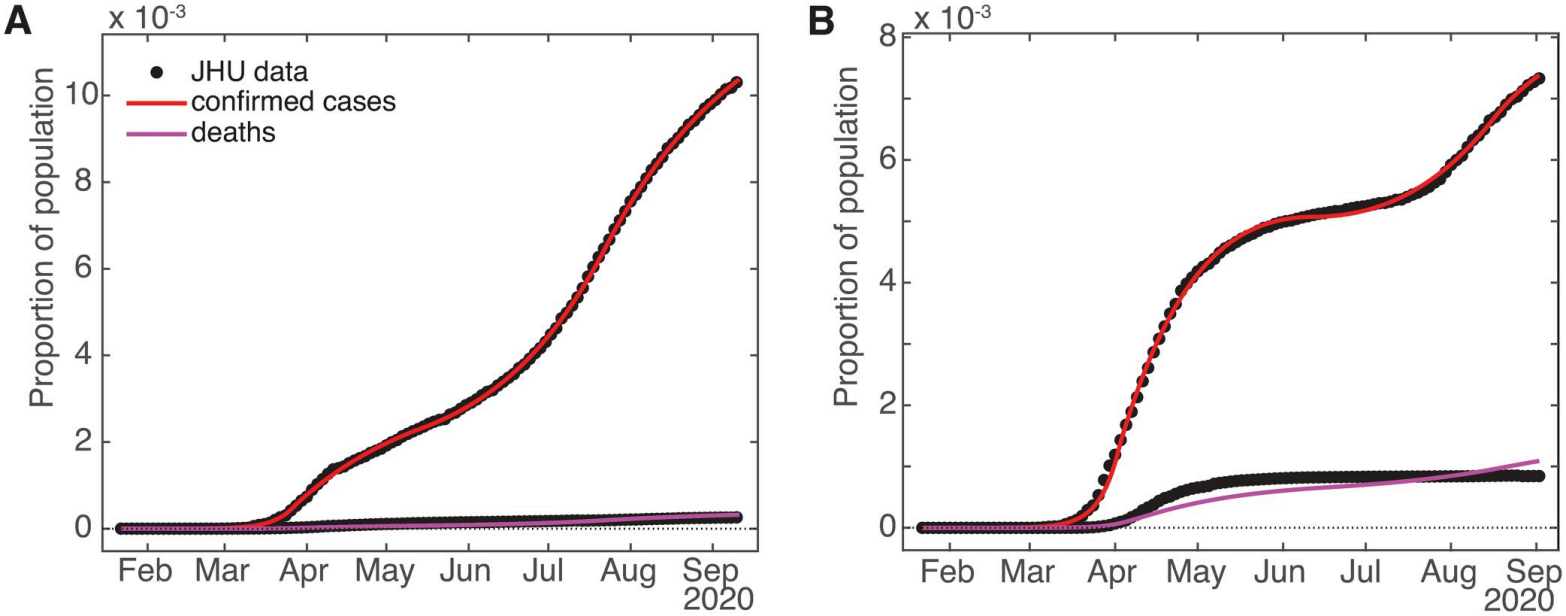
Model fits using one death rate to cumulative case and death data. (A) Washington State and (B) Belgium. These two jurisdictions were chosen at random from out of US and European datasets.

**Fig 3:**
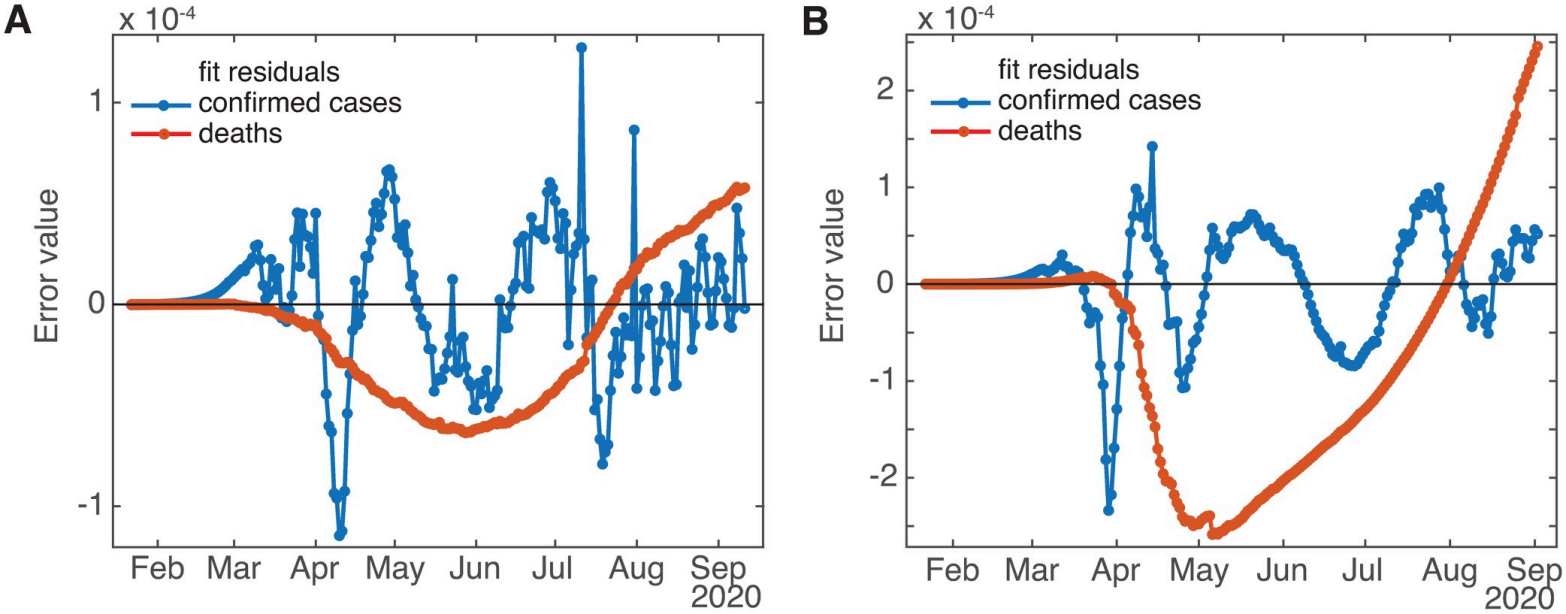
One-death-rate model fit residuals. (A) Washington State and (B) Belgium. Note distinct time-dependent bias in error for fits to death data.

**Fig 4:**
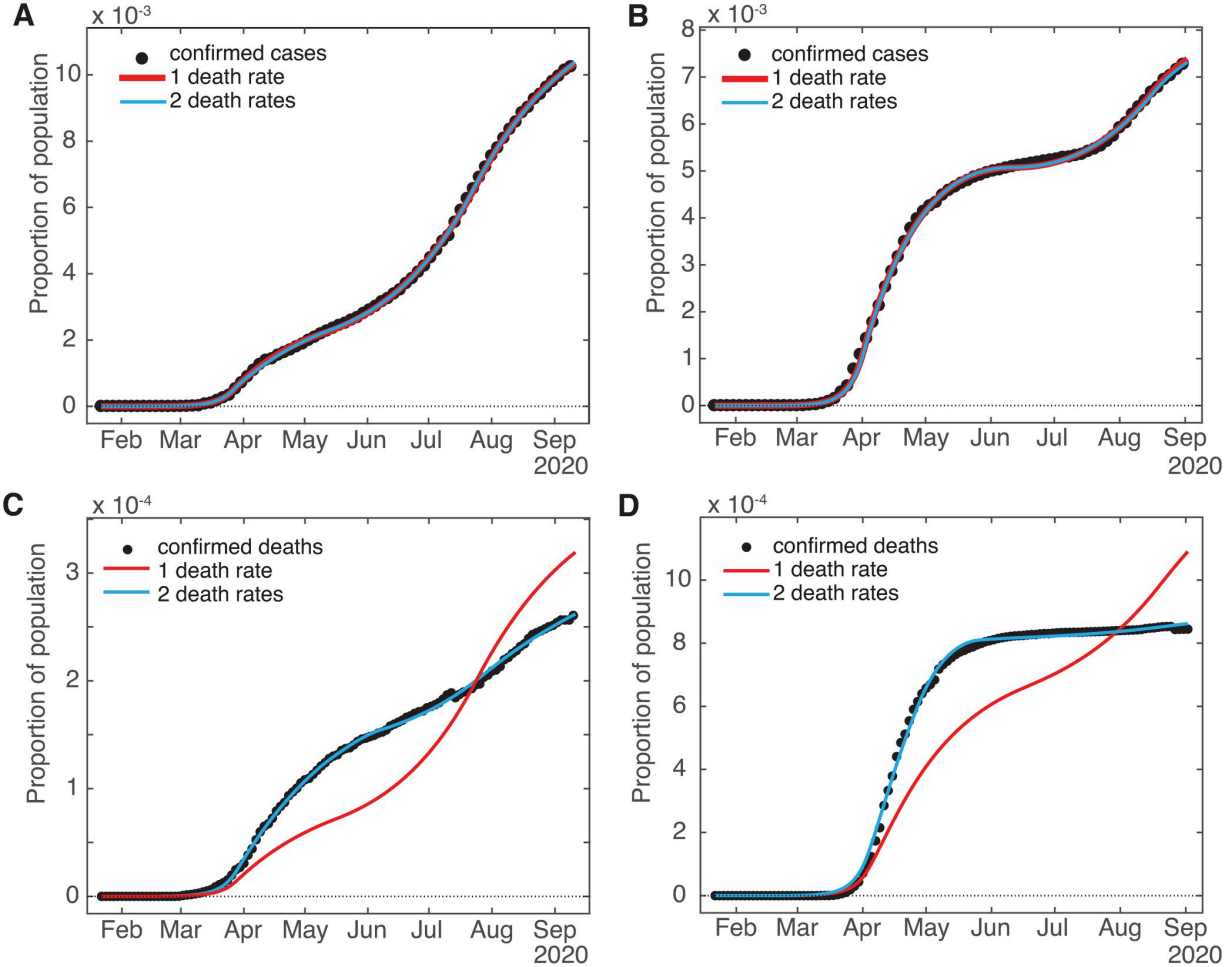
Comparison of one-death-rate to two-death-rate model fits. (Left) Washington State and (right) Belgium. (A and B) Cumulative confirmed cases, which show practically no change between versions. (C and D) Cumulative deaths.

Lastly, one assumption made in [21] that we need to change is that *γ*, *β*, and the other rates are stable, at least across the intermediate term of approximately 6 months. In particular, here we introduce to the model a bifurcated death rate (*γ*_1_ and *γ*_2_) along with a changeover date *t*_*γ*_ (increasing the number of fit parameters by two). This was made necessary by the fact that the death compartment *D* has only one incoming pathway (from the detected infecteds *I*_*D*_) and no outlet, so with a fixed *γ* the value of *D* at any time is slaved to the cumulative value of *I*_*D*_ (or equivalently, the derivative of *D* is slaved to the current value of *I*_*D*_). We found in the late summer of 2020 that the empirical curves for cumulative diagnosed cases and deaths were no longer in sync. When checking the results of 1-month-ahead death predictions, they seemed to be quite high, given that contemporary measures of deaths for various jurisdictions [9] were not showing spiking activity (though the recent new spikes in cases at the time suggested that deaths should be on the rise too). The problem was that the empirical (and therefore model) deaths were small in number compared with confirmed cases, so discrepancies between the model deaths and data were not readily apparent to visual inspection or the error criterion used by the fit routine, as can be seen from Fig 2. However, a closer look at the death curves alone revealed that while the error level was within the desired tolerance, the residuals were not (more-or-less) randomly distributed across time, but showed a distinct bias, undershooting during the first half of the fit period and overshooting at later times, so the fit curve missed the contour actually described by the death data—see Fig 3 for residuals and Fig 4(c) and 4(d) for closeups of the fits. This caused the slope of the model deaths at the end of the fit period to be greater than that implied by the empirical data, so that model projections of deaths into the fairly near future would overestimate the number significantly. Since this had not been a problem earlier, the implication was that the cumulative deaths were no longer shadowing the cumulative case count.

As one can see in Fig 4, for the time frame of the current study the assumption of 2 death rates with all other rates constant (except the sequestration variable *q*_*t*_) is sufficient for quite good fits to the empirical data. However, going forward from this time period to deal with the vaccine rollouts in 2021 as well as the onset of the more contagious Delta and Omicron variants of the virus (plus a possible second decrease in the European case mortality rate in mid-2021, though as of yet no corresponding second decrease in the US [22]) would presumable require a model that allows all the coefficients to vary with time. We are now close to completing an implementation of such a model, with which we hope to do follow-up work to this study.

### Implementation

All simulations, fits, analyses, and data management were done in Matlab using COVID-19 time-series data for cumulative confirmed cases and deaths acquired from the Johns Hopkins University (JHU) Center for Systems Science and Engineering public repository on GitHub [23]. This was the only source we used for COVID data; US state population data is from the US Census Bureau [24] (European populations were already available in the JHU tables), and the economic and demographic data used in the correlations are from various sources such as the IMF, CIA, and World Bank [25–30]. As will be discussed below, the JHU assembles its data from many official sources such as the WHO, CDC, and national and state health agencies. Raw data in the original CSV files were converted to Matlab table data structures before any processing. From the JHU national data we selected the European countries by the simple geographical criterion of the country’s central longitude and latitude, thus including Turkey but discarding Russia. Since the JSU’s US data was broken down by county, these had to be aggregated to get statewide time series. As well, data for overseas US territories other than Puerto Rico was discarded. The fit period was Jan 22 to Sept 11 for the US and Jan 22 to Sept 2 for Europe.

Coding of the model falls into three functional layers: solving for compartment derivatives at a single point in time, using the derivative solutions to solve the ODE’s over the entire time series period, and using the ODE solution to fit to empirical data to find parameter values. On the outermost level, least squares fits of normalized JHU time series for cumulative cases and deaths were done using Matlab’s lsqcurvefit, which is an iterative solver designed for non-linear problems. Fit parameters were: all fixed model rate coefficients (*β*, *α*_*U*_, *α*_*D*_, *δ*, *ρ*), the two death rates *γ*_1_ and *γ*_2_ plus the changeover time *t*_*γ*_, the power law exponent *a*, initial condition for unknown infecteds *I*_*U*_(0), plus for every imposition of or release from social distancing measures a pair *q*_*i*_, *t*_*i*_ giving the magnitude and time of application. The way our implementation is coded allows us to specify an arbitrary number of such interventions; for this project, there were three such pairs (the initial spring lockdown, a relaxation in the late spring / early summer, and a usually weaker reimposition of measures when cases started to spike again in the late summer. Since the model is non-linear, fitting requires an iterative search through the solution space, so there is no guarantee of obtaining optimal solutions within any set running time, but we found that with adjustments to the fit parameters by restarting searches, when necessary, from randomly changed points in the solution space we were able to get good fits for all US states within a couple of hours (the criterion we use for goodness of fit was that the coefficient of determination *R*^2^ > = 0.95; *R*^2^ gives a normalized measure of the proportion of variation in the empirical data that is replicated by a model). We were also able to fit all European countries Covid-19 case and death data (except Russia) using the same optimized code with similar results.

lsqcurvefit works by repeatedly invoking a user supplied target function on parameter values determined by where it is in the search space, which it tests against the given data. For the target function we used Matlab’s ode45 function to evaluate the ODE system given in Eqs 1–7 over the data’s time period with rate coefficients, exponent, and times as given by lsqcurvefit. ode45 is itself an iterative solver that at each step also invokes a user supplied function to evaluate the compartment derivatives at a specified time based on current compartment values and given parameters. Aside from the *q*_*t*_ interventions the coding for this is straightforward. The death rates *γ*_1_ and *γ*_2_ ramp linearly in a four week period centered on the changeover time *t*_*γ*_. Since the peak *q*_*t*_ rate is of little interest in its own right, for ease of analysis we decided to have the *q*_*i*_ values in the parameter set represent the final proportion of the *S* or *Q* compartment we wanted moved i.e. if *q*_*i*_ = 0.25, a quarter of *S* and *I*_*U*_ are moved to *Q*, and if *q*_*i*+1_ = −0.5, half of *Q* is moved back, so now *S* is roughly $\frac{7}{8}$ of its original size (depending on what other transfers have been occurring). The actual values of the *q*_*t*_ rate are then set so as to transfer this amount of population in approximately two days centered on the designated transfer time *t*_*i*_. For more in-depth discussion of the this and other aspects of the implementation see the first appendix in [21]; our Matlab code is freely available at https://github.com/fvbttu/squider/tree/master/code.

## Results

To obtain our results model fits were done on 52 US jurisdictions (all states, plus Washington DC and Puerto Rico), and on 49 European zone countries (i.e. all of Europe except for Russia, plus Turkey). The fit period was Jan 22 to Sept 2 for Europe and Jan 22 to Sept 11 for the US. Each fit provided the two death rates and a changeover time (in days from the start of the fit period, Jan 22 2020); percent change from *γ*_1_ to rate *γ*_2_ was calculated as well ($100\times \frac{\gamma_{2}-\gamma_{1}}{\gamma_{1}}$). See Tables 4 and 5 in the S1 Appendix for a full listing of rates, changeover day, and percent change for each US state and European country studied. Fits for all jurisdictions were redone in mid-December to confirm the results; while values for particular rates did show movement of a few percentage points (in both directions), the basic trend proved to be robust (see S2 Appendix). We doubt that extending the fit period beyond this will be of use for this particular work, since the rollout of vaccines and the arising of new variants may have confounding effects.

**Fig 5:**
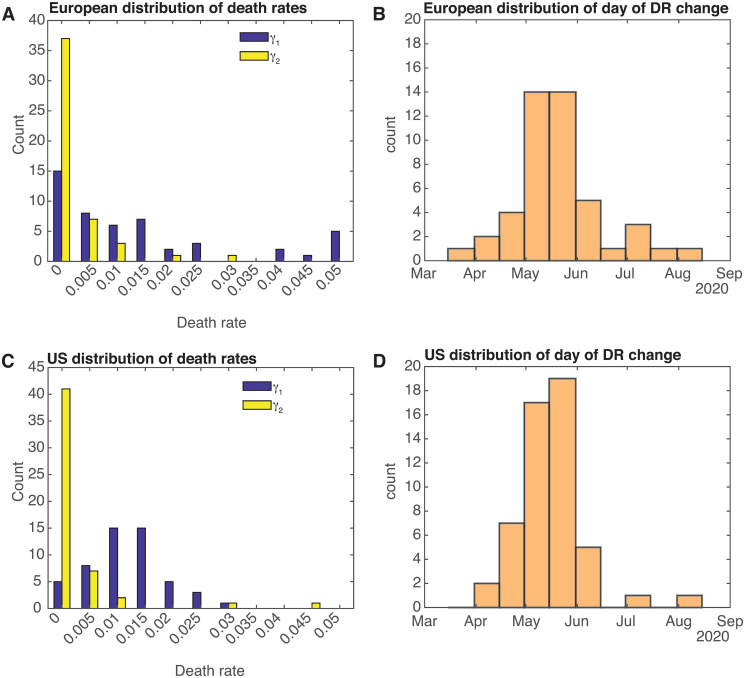
Death rate distributions. (A) Distribution of first *γ*_1_ and second *γ*_2_ death rates, and (B) distribution of the day of death rate change for all European countries (except Russia). (C) Distribution of first *γ*_1_ and second *γ*_2_ death rates, and (D) distribution of the day of death rate change for all US states.

We start with the rates themselves (see Table 1). The initial death rate for detected infecteds is approximately 1% in Europe and 1.5% in the US, which is consistent with values being hypothesized/calculated in March/April of 2020 [22]. These go to approximately 1.5 per 1000 and 3 per 1000 respectively, a 5- or 6-fold drop. The changeover time in the fit period corresponds to the dates of May 18 in Europe and May 15 in the US. As implied by the standard deviations of the changeover times (19 days in Europe and 33 days in the US), most of the drops occurred within the period between mid-April and mid-June. Fig 5 shows how all the rates and changeover times are distributed. The second death rate *γ*_2_ is much more narrowly distributed than the first death rate *γ*_1_, which, given that this cannot go below zero, is not surprising.

**Table 1:** Statistics for the death rates *γ*_1_ and *γ*_2_, as well as the date of the change *t*_*γ*_.

Metric	Europe	US
Median *γ*_1_	0.01058	0.014801
Standard deviation *γ*_1_	0.022353	0.0071521
Median Changeover *t*_*γ*_ (in days)	117.7146	114.6639
Standard deviation *t*_*γ*_ (in days)	33.1116	19.2085
Median *γ*_2_	0.0014119	0.0028296
Standard deviation *γ*_2_	0.0058485	0.0075316

### Change in death rate

**Fig 6:**
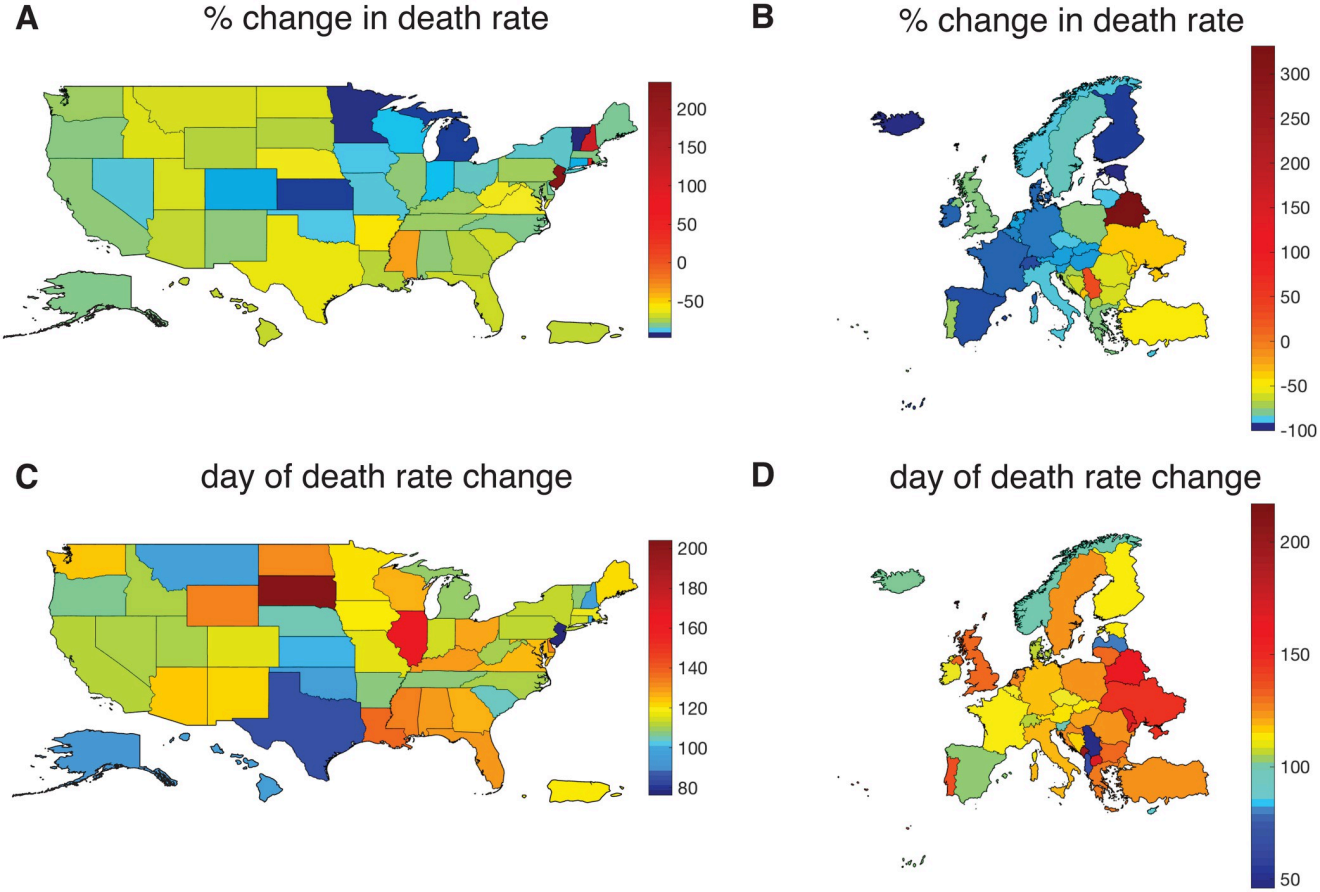
Geographical changes in Covid-19 death rates. (A and B) Percent change of death rate for US and Europe. (C and D) changeover time (in days since January 22, 2020). These maps were generated using Matlab’s Mapping Toolbox [34].

While it is to be expected that the case mortality rate of a disease will drift downward over time as medical treatments improve [31–33], both the relatively tight timing and magnitude of the change in death rates are noteworthy; we see a decrease of approximately 90% across Europe and 80% across the US within a 2-month period. Table 2 shows various statistics related to this drop. Fig 6 gives maps of the US and Europe color-coded by the drop in rates and the changeover day; in the former particularly we see that western Europe mostly saw large decreases, while eastern Europe is more variable. We also observe that US outliers with large positive changes in death rate are in the east. While there are clusters for the day of death rate change in Europe and the US, no clear pattern is apparent.

**Table 2:** Statistics for the percent change in death rate.

Metric	Europe	US	comment
# Jurisdictions	49	52	
Mean % change in death rate	-70.0085	-67.366	
Median % change in death rate	-91.0148	-80.6421	
Mode % change in death rate	-94.8642	-83.2356	
Outliers	6	3	with positive change
Greatest decrease	-100	-97.3713	
Least decrease	-38.2119	-38.0591	
Greatest increase	330.9656	234.5559	excluding countries 0 reported deaths
Standard deviation	16.6705	11.6069	(and below) excluding outliers
Skewness	1.5267	0.96071	> 0—skews right
Kurtosis	4.3511	4.5883	> 3—thicker tailed distribution than Gaussian

### Outliers

Not all jurisdictions saw decreases in death rates according to the measurement derived from out model. In the US three states, New Hampshire, New Jersey, and Rhode Island had increases, of 119, 235, and 55% respectively. New Jersey and Rhode Island had relatively late dates for the effective release from lockdown in comparison with other states, as measured by the model (June 16 and July 11 respectively). Mathematically, since these states did not open up at the same time as the others, their cases did not start rising dramatically again in the early summer, so the denominator defining the case mortality rate stayed relatively low. It should be noted that when new fits were done in December, all three states now showed decreases in the same 80–90% range as the rest of the US, though the dates associated with the changeover were significantly later than average (late June to late July).

In Europe the outliers break down into two different groups. In the first case we have the Faroe Islands, Gibraltar, and Latvia, which had effectively no deaths in the period before the measured changeover (Faroe Islands and Gibraltar apparently had no deaths whatsoever during the entire fit period); in this case the extremely large positive rate changes are an artifact of the extremely low initial rates given by the fitter. It should be noted that Latvia’s neighbor Estonia had no recorded deaths in the period after changeover, and so achieved a 100% drop; this suggests that the death statistics in the Baltic states may themselves be an issue.

The second group of European outliers—Belarus (331% increase), Kosovo (78%), and Serbia (30%), like the US outliers, are more perplexing. The latter two were notably involved in a violent conflict in the 1990’s; all three are not members of the EU. Aside from that we can note that these countries had relatively late outbreaks (with first deaths recorded on March 22, March 29, and April 28 respectively), resulting in a later surge of cases and deaths. These, like the US outliers, all showed decreases instead of increases when fits were redone in December, though in this case only to 50–60% levels. For these countries, as well as the US outliers, changes in the COVID-19 death reporting protocols may play a part in the initially measured increase becoming the same kind of decreases as seen elsewhere (see the Discussion below).

### Correlations

One may ask if there is a relation between the measured changes in death rates and various other metrics. However, with one rather trivial exception (to be discussed below) we found no strong correlations of the drop to either model-related quantities or a number of readily available state/national statistics; though admittedly, our search through national databases was not exhaustive. All correlations discussed below were calculated using Matlab’s corrcoef function, which gives the Pearson correlation coefficient $\rho_{X,Y}=\frac{\text{cov}(X,Y)}{\sigma_{X}\sigma_{Y}}$, where cov(*X*, *Y*) is the covariance of the datasets *X* and *Y*, and *σ*_*X*_, *σ*_*Y*_ are their respective standard deviations.

We started with the model parameters themselves, and quantities derived from either the raw data or projections based on the fits. Across multiple fits we would expect some rates to move in tandem or opposition to others, and indeed, for both Europe and the US we see that the SIR-based contact rate has a strong negative correlation (< −0.9) with both the recovery rate for undetected infecteds and the detection rate, as well as a slightly weaker positive correlation (> 0.67) with the severity of the first social distancing intervention. In fact, one model parameter does correlate strongly with the drop in the death rate: the second death rate itself (0.72 for Europe, 0.97 US), which is hardly surprising. However, no other rates or data derived quantities had an absolute correlation > 0.5 for either the US or Europe, and only a few had an absolute correlation > 0.33; these latter all had different signs for the European and US fits, indicating that the relation was not particularly robust despite the magnitude. Only three model related quantities other than the second death rate had absolute correlations ≥ 0.1 with same sign for the US and Europe: loss of immunity rate (negative), initial condition (proportion of population infected on day 1 of fit period, negative), and proportion of unknown recovereds on last day of fit period (positive). In all these cases the absolute correlation was < 0.22, so rather weak.

Since the correlations to standard state/national statistics may be of more general interest, these are given in Table 3. As with the model parameters, most correlations here are quite weak and have different signs between Europe and the US; only longitude has non-trivial (though not strong) correlations of the same sign, which is apparent from Fig 6(a) showing consistently larger drops in percent death rate change in Western Europe than Eastern Europe. As mentioned above, we did not check many other possible quantities (eg. educational attainment, per capita health care expenditures, etc.) since each requires finding and converting new data extraneous to our main project; in particular, certain epidemiological data, such as COVID-19 testing rate (which is itself time-varying), might yield interesting results with more intensive comparison techniques. Note that correlations between the individual death rates and changeover day with the other model parameters and state/national statistics were also calculated, and as well did not show any strong or surprising correlations (data not shown).

**Table 3:** Pearson correlation coefficients for % changes in death rates and state/national statistics. Population, area, population density, latitude, and longitude data were obtained from Johns Hopkins University alongside Covid-19 data [23].

Metric	Europe	US
Population	-0.03319	-0.045653
Area	0.057402	-0.15576
Pop. density	-0.080492	0.015033
Latitude	-0.02293	0.062915
Longitude	0.33451	0.16866
GDP [25, 26]	-0.16385	-0.05716
GDP per capita [25, 26]	-0.33194	-0.07874
Gini coefficient [27, 28]	-0.12044	0.10396
Median age [29, 30]	-0.25117	0.19846

### Counterfactual scenario

**Fig 7:**
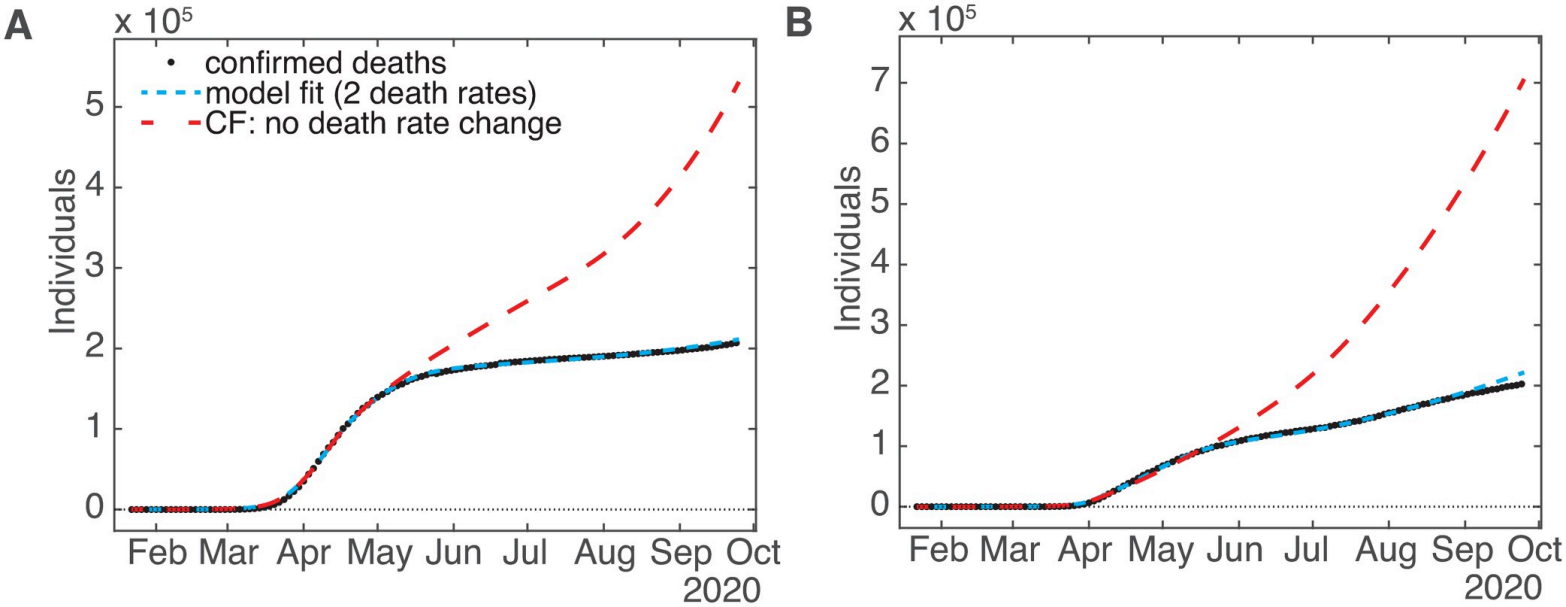
Model fit to confirmed Covid-19 deaths, and the number of deaths predicted for the counterfactual (CF) scenario of no change in death rates. (A) Europe, and (B) the United States.

Our implementation allowed us to run counterfactual simulations to test various suppositions by rerunning the ODE solver on the model with changed parameter values. By suppressing the second death rate, we are able to estimate what the deaths outcome would be if no change in rate had occurred. Fig 7 shows plots of deaths data, model fits, and counterfactual projections for Europe and the US. As one would expect, if the rate had not changed the number of deaths by mid-December 2020 would have been much greater, more than triple in the US (from ≈ 313,000 to 997,000) and more than quadruple in Europe (from ≈ 454,000 to 2,200,000). Since the effect on the cumulative confirmed cases was minimal, we have not shown these plots.

## Discussion

There are several factors expected to affect fatality rates over the course of a pandemic. A large scale study involving researchers from Croatia, Italy, Spain, the UK, China, Finland, Poland, Germany, and the US attributes the decline to seasonality [35]; while this has some plausibility, the fits to US state data we continued to do for the COVID-19 ForecastHub over the winter did not require the addition of a third death rate to maintain the desired quality. A commonly cited cause is improvements in medical treatments, which are to be expected as knowledge about the disease increases. In part this could be due to gains in efficiency as health workers become experienced with the disease; a study of Massachusetts cases between March and July found a significant decrease in the number of complications encountered [36]. As well, developing specific treatments for COVID-19 became a major focus of research. For example, aggregated data suggests that transfusing (high anti-RBD IgG titer) convalescent plasma early in the hospital course of Covid-19 patients significantly reduces mortality by approximately 6% in comparison with control patients [31]. Additional independent studies have shown that administering tocilizumab (a recombinant monoclonal antibody that can mitigate cytokine release syndrome) to patients admitted to intensive care with Covid-19 were reported to have a 23% [32] and a 12% [33] reduction in mortality, compared with patients receiving standard care. Importantly, a clinical outcomes study reported that patients who presented in hospital with sufficient vitamin D levels (≥ 30 ng/ml) had reduced mortality rates by 10% in comparison with Covid-19 patients with insufficient (< 30 ng/ml) vitamin D [37], which suggests that lowly toxic supplementation and increased sun exposure can affect a population’s outcome. While the studies above generally focus on cases requiring hospitalization, they do suggest that even for serious instances mortality has been reduced by at least 20%.

### Mask wearing

It has also been suggested that mask wearing can reduce the mortality rate of Covid-19 via two different means. First, a face mask worn by an infected person forms a barrier for transmission of respiratory droplets to susceptible populations, thus reducing transmissibility of the disease [38, 39]. It may be reasonable to expect that populations with widespread mask usage and clear government guidelines may have a reduction in contact rate *β* associated with policy implementation if the policy had been implemented after occurrence of exponential growth in cases [40]. We have not observed the need for such a reduction to fit cumulative cases in any country or state well. In any case, while reduced case counts (if we had seen them) would result in fewer deaths overall, this says nothing about deaths per case. But it is also possible that wearing a face mask protects the wearer by reducing the SARS-CoV-2 inoculum that they are exposed to by infected people [41]. Exposure to a low viral load may result in a less severe, possibly asymptomatic, infection with a lower chance of fatality [42]. So it is still possible that the changed Covid-19 death rates were have observed result from face mask wearing; the YouGov online survey reporting tool demonstrates that self-reported face mask wearing in public spaces in some European countries (Italy, Spain, France, and Germany) rapidly increased to 80% of the population or more between late March and May 2020 [43]. A similar trend is observed in the United States, where self-reported face mask wearing in public places rose to 69% at the end of May 2020 [43]. However, self-reported face mask wearing in the Nordic nations of Finland, Denmark, Norway, and Sweden did not exceed 20% of the population over the same time frame, and these nations also experienced very large drops in death rate. This evidence strongly suggests that if wearing face masks is a factor that affects the death rate change, it is not the only one.

### Variant strains

It is also possible for a virus to acquire mutations that alter its infectivity and lethality over time. Genomic analyses have demonstrated that the spike protein of SARS-CoV-2 had undergone an amino acid change from aspartic acid (referred to as D614) to glycine (referred to as G614) in the carboxy-terminal region of the S1 domain [44–46]. The very rapid spread of the G614 mutant throughout Europe and parts of the Americas in 2020, monitored by Covid-19 genetic surveillance studies over time, suggests that it could be more transmissible [44–47]. One regional study conducted within a Houston hospital system showed that the virus strains originally introduced into the city in March 2020 were diverse, with both D614 and G614 types represented; however, sequences taken during the much larger second wave that occurred in June 2020 were nearly all of the G614 type [48]. They found that patients with the G614 strains had higher nasopharyngial viral loads upon diagnosis; however, the authors did not find evidence connecting virus genotype with altered virulence [48]. Interestingly, a data correlation study found that the G614 mutation corresponds to higher case fatality rates in several countries [49]. Given the available evidence, it seems likely that the highly prevalent G614 mutation is not less deadly than previous strains. Several new variants from the UK, South Africa, Brazil and elsewhere appeared in late 2020 which appear to be more contagious then the earlier variants [50], but whether they are less or more deadly once contracted lies outside of the timeframe of this study.

### Testing

Increasing testing could also significantly impact the case fatality rate of a disease, since detecting increasing numbers of cases will increase the denominator of the case fatality rate, and possibly lead to earlier detection of a disease leading to earlier treatment thereby also reducing mortality [51, 52]. Indeed, one Italian study examined changes in the CFR in the first 50 days of the pandemic, and found that the CFR increased along with the percentage of positive tests until March 25, 2020 when the number of tests performed significantly increased [53]. The authors’ new metric, the expected CFR (obtained from interpolated logistic model fit parameter ratios from case and death curves) plateaued and trended downward from the 40th day of the epidemic. In the last ten days. both expected CFR and percentage of positive tests decreased, suggesting that improved epidemiologic surveillance resulted in fewer deaths (which were reduced by approximately 1%). Note that this trend was not found with the CFR [53]. Also, several studies examining regional differences in fatality and testing were done.

One study comparing USA, Italy, UK, France, Spain, Sweden, and Germany found that case fatality rates, normalized by the ratio of tests to total number of positive cases, tended to cluster, suggesting a correlation between mortality and testing rate [51]. Another study, using Spearman correlation coefficients, found that testing coverage (the number of tests per confirmed case), but not the total number of tests performed, was highly correlated with both population mortality and case fatality rate for 36 Organization for Economic Development countries (which include the US, UK, and many European Countries) and Taiwan [54]. In comparison, a multivariable statistical study of Covid-19 mortality in 196 countries found that a 10 times decrease in per-capita testing was correlated with a 26% increase in per-capita mortality, though this correlation was not found to be statistically significant [52].

Another statistical comparison of testing rates and mortality across French region borders found that performing an additional 2000 tests would save three lives [55]. Data available from the Johns Hopkins University Coronavirus Resource Center [56] shows that US tests increased 12 times (from 0.1 to 1.2 million) from April through November 2020, and data from Our World in Data shows that US tests per case increased from 4.9 on April 2, 2020 to 13.3 on November 1, 2020 [57], suggesting that increased testing played a role in the large death rate decrease we have observed in nearly all US states. Similarly, tests per 1000 people in European countries ranged from 0.9–3.2 on April 1, 2020, whereas they increased to 1.99–17.25 on November 1, 2020, and the tests per case ranged from 4.5–33.3 in European countries on April 1, 2020, and ranged from 3.2–57.9 on November 1, 2020 [57], also suggesting that increased testing may have reduced Covid-19 CFR in European countries. Note that our death rates cannot be correlated with daily testing or tests per case since we are not measuring a daily death rate.

### Demographics

It is also possible that the age demographics of people more recently afflicted with Covid-19 have affected the mortality rate—particularly if more young people than elderly have become infected—who tend to be much less likely to have severe disease [58]. Indeed, an analysis of Covid-19 cases that occurred worldwide between February and July, 2020 revealed that the number of infected people 15–24 years old increased from 5% to 15%. Cases of Covid-19 in the USA in people 18–22 years old increased by 55% from August 2-Sept 5, 2020, and was highest among people between 20 and 29 years old, with more than 20% of the total cases, in contrast with March 2020 where Covid-19 incidence was highest in people with ages over and including 60 years [59]. The implication that demographic shift is responisible for the trend was reinforced by an Italian study showing that age-specific mortality did not change significantly during the first six months of the pandemic [60]. However, other clinical reports do indicate that Covid-19 has become less deadly across all age groups. It was reported that the mortality rate, adjusted for changes in demographics, had dropped by 18% in a New York city hospital system from March to August 2020 [12]. Similarly, English hospital surveillance data found that the survival of Covid-19 patients in both intensive care and high intensive units increased by approximately 11% per week from late March through June 2020 across age, ethnicity, and major co-morbidity subgroups [13]. A larger scale statistical survey covering 12 European countries, the US, and Japan later in the year found that nursing home deaths due to Covid-19 in the second wave of the pandemic decreased significantly while the age distribution of deaths between the first two waves stayed roughly the same [61]. Given these varying results, it appears that changes in age demographics of Covid-19 incidence cannot fully explain our observed change in mortality over time.

### Data collection and reporting

Lastly, we look at the possibility that the drop could be a statistical artifact caused by changes in the way death data is recorded and collected. It should be noted, that we (along with [9]) first noticed the change of death rate not as a drop in daily deaths versus total population, but as persistence of the previous trend when surges in the number of cases versus total population occurred after releases of lockdowns, where concomitant surges in deaths were to be expected.

Data revision is common for many publicly maintained statistics, not only in medical areas but also economics and demographics, since later figures often improve or correct earlier ones, which may be based partly on estimates or incomplete surveys. With respect to diseases or mortality, large upward revisions often gain public attention, since the implication is of prior negligence or coverup. Examples include China’s April 2020 revision upward by 1290 deaths (which increased their case mortality at the time by 50%) [62], and Argentina’s large upward correction at the beginning of October 2020 [63].

There are legitimate reasons for changes in procedure that result in lower death counts and subsequent downward revisions. Many jurisdictions initially logged all deaths of Covid-19 infected individuals as deaths by Covid, presumably because in the early days of the pandemic the exact range of co-morbidities had not been determined; when later information is available to limit that range, non-Covid deaths of Covid-infected individuals can be placed in the appropriate category. This is the case for the UK revision in August 2020. Previously, the UK had been counting all deaths of Covid-infected people within 60 days as death by Covid-19, which was reduced to 28 days; applied retroactively, this had the effect of reducing the UK Covid-19 death count by 5,377 (≈ 13% at the time) [64]. Similarly, Washington State, which had been counting all deaths of anyone who tested positive at any point as Covid-19 deaths, officially adapted a more stringent protocol in mid-June 2020, only listing a death as Covid-related if it was a specific factor mentioned in the death certificate [65]. Case and death reductions may also occur for other reasons. In Belgium a downward revision, ostensibly to correct for double-counting in nursing homes, made news because it seemed to be timed to avoid the milestone of 10,000 Covid-19 deaths [66].

Downward revisions of past death statistics, if integrated properly into time-series data, should not have an adverse affect on any attempt to determine changes in case-mortality over time, whether by our model or other techniques. Our primary data source, the JHU CSSE Covid team [23], seems to have made every effort to revise past data to reflect current knowledge and practice. To begin with, they cross-reference many sources of their own, including the World Health Organization, the European Centre for Disease Prevention and Control (ECDC), the US Center for Disease Control, many other national health organizations (such as the National Health Commission of the People’s Republic of China, Australia Government Department of Health, Italian Ministry of Health, etc.), practically all US state Departments of Health, many municipalities and US counties, news organizations such as the Los Angeles Times and BNO News, and even a few other Covid-19 tracking projects (presumably for confirmation) such as “the COVID Tracking Project” maintained by The Atlantic (https://covidtracking.com/data) and WorldoMeters Covid page (https://www.worldometers.info/coronavirus/).

Importantly, when possible the JHU CSSE Covid team back-distribute revisions of past data (i.e. incorporate them on appropriate days in their currently available time series). According to their records, there have been 22 data modifications for European nations and 19 for US jurisdictions (which are tallied by county). As well, several large-scale back distributions have been done (twice for both New York City and Massachusetts; and once for the United Kingdom, Michigan, New Jersey, North Carolina, and Harris County, Texas). In general, such back distribution (whether an up or down revision) should make death data before mid-May 2020 more trustworthy rather than less.

An issue arises if jurisdictions adopt new protocols without revising past statistics, or do the revisions without back-distributing into the past data sets. In the JHU CSSE time series we used, 36 US states and 21 European countries had decreases in cumulative deaths on 121 separate occasions, mostly by 1 or 2 cases. Since any decrease in cumulative deaths is a physical impossibility, the ones we see here presumably indicate data revisions which could not be back-distributed. For example, the time-series we used for Washington State has occasional negative day-to-day changes in death counts starting from mid-June 2020 (when they changed their protocol) and lasting through July 2020 (when they seem to have finished whatever revisions they needed to make). The total number of deaths involved in these post hoc revisions is 2,463 for the European nations and 666 for the US states; while not trivial, these values could hardly account for the drops we have seen in the death rates detailed above. To determine how many downward non-back-distributed revisions occurred which did not result in negative day-to-day changes in cumulative deaths, or which countries, states, or counties quietly adopted different protocols or definitions without attempting to revise past totals, would require greater access to jurisdictional health agency revision and policy data than we have.

## Conclusion

In conclusion, while there are many plausible factors, such as improved medical techniques, mask wearing, increased testing, viral mutation, demographics, or changes in recording of cases, that may have caused the dramatic decrease of the case mortality rate of Covid-19 we have measured in the US and Europe, at this point we cannot conclusively say which, if any, are *the* cause, or if it is a combination of these or other subtle factors. However, there can be no doubt that this phenomenon is distinct from the gradual downward drift in death rate one might expect as the medical community comes to consensus on best practices while both the human and microorganism populations adjust and evolve into a more livable coexistence.

Additionally, one may ask whether the lower death rates current in the US and Europe will persist. Fits going to the end of 2020 seem to show that the decrease in rate is stable; however, new COVID-19 variants that emerged in the U.K., South Africa, and Brazil [50], as well as the various vaccine rollouts across the globe, might change that. While it would be simple enough to code a 3-death-rate model based on our current model, the aforementioned factors would also affect transmission, duration, and possibly even detection of the disease; hence we have begun to look into using a general multi-rate model for fitting. Preliminary work indicates that such a model does not pose any problems with respect to solving the ODEs; because of the increase in number of the dimensions of the solution space, fitting raises some issues, but these can likely be dealt with by doing the fits in stages.

## Supporting information

S1 Appendix (PDF)

S2 Appendix (PDF)
