# Supplementary material for: Modeling the change in European and US COVID-19 death rates
Source: PLoS One. 2022 Aug 17;17(8):e0268332. doi: 10.1371/journal.pone.0268332 (PMC9385065; doi:10.1371/journal.pone.0268332)
Supplement: S1 Appendix — (PDF) [file pone.0268332.s001.pdf]

Appendix I

Table 4. Death rates γ_1 and γ_2, day of change t_γ and corresponding date, with percent change for US states.

**State**	γ_1	t_γ	**Date (2020)**	γ_2	**% Change**
Alaska	0.018858	91.9803	Apr 22	0.0029998	-84.0922
Alabama	0.01584	129.1083	May 29	0.0028764	-81.8412
Arkansas	0.01501	107.0161	May 07	0.006258	-58.3066
Arizona	0.019334	122.9221	May 23	0.005112	-73.5597
California	0.014878	111.098	May 11	0.0026375	-82.2724
Colorado	0.013955	115.4642	May 15	0.0010777	-92.2773
Connecticut	0.0077278	113.8637	May 14	0.0006074	-92.14
District of Columbia	0.01744	126.0829	May 26	0.0014485	-91.6943
Delaware	0.01066	132.3503	Jun 01	0.001541	-85.5436
Florida	0.015309	129.4741	May 29	0.0042771	-72.0615
Georgia	0.011839	125.6616	May 26	0.0028935	-75.5597
Hawaii	0.0092434	94.9948	Apr 25	0.0024744	-73.231
Iowa	0.0041224	119.2705	May 19	0.00037223	-90.9704
Idaho	0.014863	110.389	May 10	0.0047981	-67.7173
Illinois	0.023902	164.9266	Jul 04	0.0043251	-81.9046
Indiana	0.01642	113.6621	May 14	0.0013726	-91.6409
Kansas	0.0066308	99.5549	Apr 30	0.00028062	-95.768
Kentucky	0.028502	128.7954	May 29	0.0057361	-79.8748
Louisiana	0.026275	137.2672	Jun 06	0.0064757	-75.3538
Massachusetts	0.0053833	117.0038	May 17	0.00089078	-83.4529
Maryland	0.0052426	124.0183	May 24	0.00098801	-81.154
Maine	0.013773	120.8261	May 21	0.0020725	-84.9523
Michigan	0.022984	108.211	May 08	0.00097971	-95.7374
Minnesota	0.012611	120.2323	May 20	0.0004156	-96.7044
Missouri	0.019139	115.7427	May 16	0.0019625	-89.7462
Mississippi	0.017016	133.2993	Jun 02	0.01054	-38.0591
Montana	0.010594	96.861	Apr 27	0.0031836	-69.9485
North Carolina	0.01474	110.1662	May 10	0.0023085	-84.3386
North Dakota	0.018339	131.7428	Jun 01	0.0057475	-68.6592
Nebraska	0.012986	104.1514	May 04	0.0046664	-64.065
New Hampshire	0.014292	96.4596	Apr 26	0.031264	118.7539
New Jersey	0.014136	76.3807	Apr 06	0.047291	234.5559
New Mexico	0.01693	121.5203	May 22	0.0029829	-82.3808
Nevada	0.027147	110.4525	May 10	0.0027828	-89.7491
New York	0.034929	112.4836	May 12	0.0039349	-88.7347

Continued ...

State	γ_1	t_γ	Date (2020)	γ_2	% Change
Ohio	0.010123	127.3317	May 27	0.001216	-87.9875
Oklahoma	0.024237	95.1332	Apr 25	0.002249	-90.7209
Oregon	0.01994	105.7741	May 06	0.0033566	-83.1668
Pennsylvania	0.0042774	112.0262	May 12	0.00088788	-79.2426
Puerto Rico	0.015866	119.5141	May 20	0.0041358	-73.933
Rhode Island	0.0074883	99.257	Apr 29	0.011599	54.897
South Carolina	0.01325	103.3883	May 03	0.0038586	-70.8792
South Dakota	0.0064334	203.8722	Aug 12	0.0015365	-76.1174
Tennessee	0.0061235	107.274	May 07	0.0012167	-80.1301
Texas	0.011842	84.0796	Apr 14	0.0041029	-65.3542
Utah	0.0037314	110.1407	May 10	0.0011337	-69.6166
Virginia	0.017346	124.8077	May 25	0.0066082	-61.9042
Vermont	0.023989	107.7641	May 08	0.0006306	-97.3713
Washington	0.023503	123.4539	May 23	0.0047135	-79.9452
Wisconsin	0.0044732	125.8417	May 26	0.00037944	-91.5175
West Virginia	0.019036	109.8525	May 10	0.0069387	-63.5491
Wyoming	0.0015296	132.9206	Jun 02	0.00036233	-76.3115

Table 5. Death rates γ_1 and γ_2, day of change t_γ and corresponding date, with percent change for European countries. * for change indicates country had no deaths before time t_γ.

Country	γ_1	t_γ	Date (2020)	γ_2	% Change
Albania	0.046842	62.1243	Mar 23	0.0080575	-82.7986
Andorra	0.020542	116.5576	May 17	0.00055115	-97.3169
Austria	0.0098244	113.6568	May 14	0.00063225	-93.5645
Belarus	0.0047253	161.9287	Jul 01	0.020364	330.9656
Belgium	0.027217	112.0309	May 12	0.0014119	-94.8126
Bosnia and Herzegovina	0.01543	115.5272	May 16	0.005373	-65.1794
Bulgaria	0.0052442	131.303	May 31	0.0018924	-63.9136
Channel Islands	0.0078607	106.7912	May 07	0.00012372	-98.4261
Croatia	0.0081174	124.9307	May 25	0.0021014	-74.112
Cyprus	0.015286	85.6981	Apr 16	0.0014654	-90.4136
Czechia	0.0084008	111.5784	May 12	0.00078631	-90.6401
Denmark	0.023793	106.2476	May 06	0.00098962	-95.8406
Estonia	0.0024383	112.7541	May 13	4.3292e-14	-100
Faroe Islands	8.2776e-11	216.8633	Aug 25	1.9168e-09	*
Finland	0.0022821	112.5707	May 13	3.8419e-05	-98.3165
France	0.062387	112.4251	May 12	0.0024589	-96.0586
Germany	0.010068	117.7146	May 18	0.0004936	-95.0975
Gibraltar	4.1871e-14	216.2532	Aug 24	0.00062895	*
Greece	0.0095689	128.07	May 28	0.0018301	-80.8742
Hungary	0.053932	121.291	May 21	0.0033608	-93.7685
Iceland	0.0034045	100.8264	May 01	3.0382e-08	-99.9991
Ireland	0.01058	110.7097	May 11	0.00041922	-96.0377
Italy	0.055973	118.6296	May 19	0.0056654	-89.8783
Kosovo	0.0021242	178.0724	Jul 17	0.0037917	78.4974
Latvia	1.0121e-08	79.0803	Apr 09	0.031484	*
Liechtenstein	0.0044271	94.2358	Apr 24	1.9343e-08	-99.9996
Lithuania	0.014566	131.6754	Jun 01	0.001285	-91.1785
Luxembourg	0.0089991	117.8828	May 18	0.00082332	-90.851
Malta	0.0012141	130.8727	May 31	8.8242e-05	-92.7317
Isle of Man	0.019654	134.6321	Jun 04	0.00042544	-97.8354
Moldova	0.01743	168.4762	Jul 07	0.0096963	-44.3701

Continued ...

**Country**	γ_1	t_γ	**Date (2020)**	γ_2	**% Change**
Monaco	0.010934	103.4746	May 03	4.7518e-14	-100
Montenegro	0.0041409	199.6697	Aug 08	0.0025586	-38.2119
Netherlands	0.026892	125.8466	May 26	0.0017659	-93.4335
North Macedonia	0.0029871	170.4689	Jul 09	0.00082065	-72.5268
Norway	0.0014174	98.9619	Apr 29	0.00013942	-90.1642
Poland	0.016209	123.6755	May 24	0.0031553	-80.5332
Portugal	0.016931	137.2254	Jun 06	0.003978	-76.5041
Romania	0.027858	123.7185	May 24	0.010543	-62.1555
Serbia	0.011069	46.1611	Mar 07	0.014431	30.3728
Slovakia	0.0019954	113.4444	May 13	0.00013117	-93.4265
Slovenia	0.0028753	97.7511	Apr 28	0.00018597	-93.5319
San Marino	0.12548	77.8736	Apr 08	0.008933	-92.8811
Spain	0.044385	102.3834	May 02	0.0012682	-97.1427
Sweden	0.040327	123.7356	May 24	0.0051592	-87.2067
Switzerland	0.013404	105.5814	May 06	0.00035987	-97.3152
Turkey	0.0079824	124.1928	May 24	0.0039202	-50.8902
United Kingdom	0.051358	131.4971	May 31	0.0098487	-80.8233
Ukraine	0.017525	147.2525	Jun 16	0.010609	-39.4664
