# Supplementary material for: Modeling the change in European and US COVID-19 death rates
Source: PLoS One. 2022 Aug 17;17(8):e0268332. doi: 10.1371/journal.pone.0268332 (PMC9385065; doi:10.1371/journal.pone.0268332)
Supplement: S2 Appendix — (PDF) [file pone.0268332.s002.pdf]

Appendix II

Table 6. Results for US states using December 18 2020 fits.

**State**	γ_1	t_γ	**Date (2020)**	γ_2	**% Change**
Alaska	0.002256	95.2941	Apr 25	0.00018735	-91.6955
Alabama	0.016429	137.1862	Jun 06	0.0052424	-68.0897
Arkansas	0.0016304	104.6354	May 05	0.00036204	-77.7946
Arizona	0.0031288	147.334	Jun 16	0.00089023	-71.5469
California	0.0030916	135.5123	Jun 05	0.00080089	-74.0948
Colorado	0.0052972	135.9954	Jun 05	0.00074114	-86.0087
Connecticut	0.019289	156.6214	Jun 26	0.0020152	-89.5528
District of Columbia	0.017025	162.4664	Jul 01	0.0033565	-80.2844
Delaware	0.013782	189.7542	Jul 29	0.0029117	-78.8736
Florida	0.0025863	131.8848	Jun 01	0.00067164	-74.0306
Georgia	0.0047509	118.3415	May 18	0.001341	-71.7736
Hawaii	0.0016005	90.4051	Apr 20	0.00053971	-66.2787
Iowa	0.02668	156.1412	Jun 25	0.0084203	-68.4402
Idaho	0.0090478	102.9493	May 03	0.0022935	-74.6512
Illinois	0.0036423	130.5728	May 31	0.00065957	-81.8916
Indiana	0.0045881	125.7732	May 26	0.00058318	-87.2893
Kansas	0.0045097	90.6789	Apr 21	0.00015292	-96.609
Kentucky	0.0030905	126.4814	May 26	0.00031109	-89.934
Louisiana	0.0073685	127.2761	May 27	0.0017101	-76.7923
Massachusetts	0.0034515	120.4781	May 20	0.00056858	-83.5266
Maryland	0.0058165	130.2101	May 30	0.0010279	-82.3273
Maine	0.0064777	128.5356	May 29	0.001678	-74.0953
Michigan	0.012852	129.1	May 29	0.0015559	-87.8938
Minnesota	0.046884	148.3669	Jun 17	0.0078094	-83.3433
Missouri	0.0037714	128.391	May 28	0.0005042	-86.6312
Mississippi	0.024925	131.657	Jun 01	0.0093737	-62.3922
Montana	0.014598	199.5909	Aug 08	0.0087977	-39.7336
North Carolina	0.0060383	115.5982	May 16	0.0018218	-69.8298
North Dakota	0.01553	111.865	May 12	0.0077605	-50.0276

Continued ...

State	γ_1	t_γ	Date (2020)	γ_2	% Change
Nebraska	0.0084108	177.4943	Jul 16	0.0048769	-42.016
New Hampshire	0.0030749	159.6452	Jun 29	0.0006366	-79.2968
New Jersey	0.011397	185.9447	Jul 25	0.0011205	-90.1685
New Mexico	0.0029722	137.8985	Jun 07	0.00070065	-76.427
Nevada	0.0033886	133.1472	Jun 02	0.00065621	-80.6347
New York	0.023657	145.6135	Jun 15	0.0025542	-89.2033
Ohio	0.0027039	143.4198	Jun 12	0.00030024	-88.8962
Oklahoma	0.096076	108.9945	May 09	0.0072779	-92.4249
Oregon	0.0035386	117.1622	May 17	0.00079487	-77.5374
Pennsylvania	0.0053939	144.6119	Jun 14	0.0011026	-79.5587
Puerto Rico	0.077261	124.3789	May 24	0.0081087	-89.5048
Rhode Island	0.0057029	185.8076	Jul 25	0.0011704	-79.477
South Carolina	0.0042386	129.2712	May 29	0.0014303	-66.255
South Dakota	0.00041929	166.7909	Jul 06	0.00049036	16.951
Tennessee	0.0084518	68.1282	Mar 29	0.0060997	-27.8296
Texas	0.0016175	245.2861	Sep 22	0.00093977	-41.9011
Utah	0.00723	214.7449	Aug 23	0.0031431	-56.5268
Virginia	0.016927	129.691	May 30	0.0069784	-58.7744
Vermont	0.0083856	104.4703	May 04	0.0011076	-86.7915
Washington	0.008228	131.3998	May 31	0.0014154	-82.7983
Wisconsin	0.044173	125.6199	May 26	0.0070169	-84.1147
West Virginia	0.002437	111.4774	May 11	0.00059362	-75.641
Wyoming	0.0015262	126.1572	May 26	0.00079154	-48.1364

Table 7. Results for European nations using December 18 2020 fits.

Country	γ_1	t_γ	Date (2020)	γ_2	% Change
Albania	0.064042	85.6548	Apr 16	0.018712	-70.7824
Andorra	0.017761	152.4866	Jun 21	0.0012895	-92.7401
Austria	0.00564	58.5139	Mar 20	0.00051903	-90.7972
Belarus	0.0013769	65.7809	Mar 27	0.001076	-21.8479
Belgium	0.017451	102.9035	May 03	0.00074901	-95.708
Bosnia and Herzegovina	0.03823	71.988	Apr 02	0.019962	-47.7843
Bulgaria	0.017611	38.5694	Feb 29	0.001722	-90.2219
Channel Islands	0.0058546	176.093	Jul 15	3.3221e-05	-99.4326
Croatia	0.0089558	84.0945	Apr 14	0.0089861	0.33851
Cyprus	0.016124	103.3768	May 03	0.0028809	-82.1326
Czechia	0.0057004	59.1649	Mar 20	0.00054603	-90.4211
Denmark	0.030645	141.3469	Jun 10	0.0020577	-93.2854
Estonia	0.0027008	114.9705	May 15	0.00051381	-80.976
Faroe Islands	2.5173e-14	312.9255	Nov 29	2.5736e-09	*
Finland	0.0027678	114.4694	May 14	0.00022903	-91.725
France	0.04151	119.3448	May 19	0.0023776	-94.2721
Germany	0.014961	162.5469	Jul 02	0.0036738	-75.4444
Gibraltar	0.00057788	288.4433	Nov 04	0.015618	*
Greece	0.005241	34.7409	Feb 25	0.0013441	-74.3542
Hungary	0.20959	111.955	May 12	0.01503	-92.8292
Iceland	0.00048944	64.3924	Mar 25	0.00025988	-46.9023
Ireland	0.0092017	171.2454	Jul 10	0.00084106	-90.8598
Italy	0.073056	146.722	Jun 16	0.0073824	-89.8949
Kosovo	0.005126	244.3429	Sep 21	0.0026615	-48.0786
Latvia	0.01236	209.7618	Aug 18	0.011789	-4.6251
Liechtenstein	0.0031112	292.4917	Nov 08	0.011854	281.008
Lithuania	0.37802	48.6513	Mar 10	0.0074336	-98.0336

Continued ...

**Country**	γ_1	t_γ	**Date (2020)**	γ_2	**% Change**
Luxembourg	0.011732	125.1	May 25	0.0035339	-69.8776
Malta	0.0046455	260.257	Oct 07	0.01068	129.8941
Isle of Man	0.0074404	119.8415	May 20	0.0029172	-60.7924
Moldova	0.043347	138.2825	Jun 07	0.010612	-75.5189
Monaco	0.0083454	104.4866	May 04	3.1121e-11	-100
Montenegro	0.01051	209.9445	Aug 18	0.015517	47.6479
Netherlands	0.028577	121.8468	May 22	0.0012379	-95.668
North Macedonia	0.0048377	175.9706	Jul 15	0.0023598	-51.2211
Norway	0.0013265	102.433	May 02	0.00015467	-88.3404
Poland	0.015729	127.0211	May 27	0.0058486	-62.8174
Portugal	0.019345	136.6222	Jun 06	0.005647	-70.8091
Romania	0.035772	193.357	Aug 01	0.012438	-65.2307
Serbia	0.0025446	218.291	Aug 26	0.00094775	-62.754
Slovakia	0.00075475	65.1775	Mar 26	0.0009036	19.721
Slovenia	0.0084428	94.458	Apr 24	0.0023891	-71.7022
San Marino	0.034301	77.7906	Apr 08	0.0013748	-95.992
Spain	0.026784	115.43	May 15	0.0024864	-90.717
Sweden	0.04347	131.5167	Jun 01	0.0021453	-95.0648
Switzerland	0.0091089	80.1081	Apr 10	0.00074023	-91.8735
Turkey	0.0087719	308.152	Nov 24	0.00081736	-90.682
United Kingdom	0.030523	131.4732	May 31	0.002341	-92.3303
Ukraine	0.017525	147.2525	Jun 16	0.010609	-39.4664
